# Biopolymers in Nanocoating: Principles and Applications

**DOI:** 10.3390/foods15101683

**Published:** 2026-05-12

**Authors:** Vimala S. K. Bharathi, Digvir S. Jayas

**Affiliations:** 1 Office of the President, University of Lethbridge, 4401 University Drive West, Lethbridge, AB T1K 3M4, Canada; vimala.bharathi@outlook.com; 2 Department of Biosystems Engineering, University of Manitoba, E2-376 EITC, 75 Chancellors Circle, Winnipeg, MB R3T 5V6, Canada

**Keywords:** biopolymers, nanocoating, edible coating, food packaging, chitosan, cellulose nanomaterials, antimicrobial, shelf life, food safety

## Abstract

Nanocoating technology has emerged as a transformative strategy for enhancing the functional properties of food materials, packaging substrates, and food contact surfaces. This review explores the role of biopolymers as coating materials in nanocoating applications, with a particular focus on the food sector. Inorganic nanomaterials such as silver, titanium dioxide, zinc oxide, and silicon dioxide have been extensively studied for their antimicrobial, photocatalytic, and barrier-enhancing properties; however, concerns regarding toxicity and regulatory compliance continue to limit their direct food contact applications. Biopolymer-based nanocoatings present a safer and more sustainable alternative, offering biodegradability, biocompatibility, and GRAS (Generally Recognized as Safe) status. Key application areas reviewed include edible coatings for fresh and minimally processed fruits, vegetables, meat, cheese, and mushrooms; nanocoating of paper-based and polymeric packaging materials to improve gas barrier, mechanical, moisture resistance, and antimicrobial properties; nanocoating of glass or metal containers and active packaging systems, and nanocoating of food contact surfaces to prevent biofouling and microbial contamination. Recent studies confirm that biopolymer-based nanocoatings, particularly those based on chitosan, cellulose nanofibers, and alginate, can significantly extend shelf life, reduce weight loss, retard oxidation, and maintain sensory quality. Migration of nanomaterials from coatings into food systems is identified as a key safety concern. Challenges including scalability, coating durability, substrate compatibility, and incomplete toxicological profiling are critically discussed. This review underscores the need for standardized testing protocols, comprehensive regulatory frameworks, and continued research into durable, food-grade biopolymer nanocoatings as viable replacements for conventional synthetic coating systems in food preservation and packaging.

## 1. Introduction

Coating is an age-old concept that has been in use since ancient times in the food sector. Edible coating for food materials has been explored with the use of several edible polysaccharides, proteins and lipids [1,2,3]. Wax coating for fruits like apples and mangoes is commercially adopted to enhance the shelf life and the visual characteristics of the fruits. Edible coatings can also be used to prevent moisture loss from high moisture foods by acting as a barrier to the outside environment. These kinds of coatings also enhance the handling characteristics and mechanical integrity of the food material that is coated.

In addition to the materials discussed above, plant-based derivatives like peel extract, aloe gel, and mucilage can also be used as a food-grade edible coating of fruits and vegetables [4,5]. Furthermore, essential oils from various sources are also incorporated into edible coating to explore various functional properties [6,7,8]. A number of research studies have proven the potential use of chitosan [9,10,11,12,13], alginate [14,15,16,17], gelatin [18,19,20,21], pectin [22,23,24], zein [25,26,27,28], whey protein [29,30,31], and soy protein [32,33] as coating materials for food, alone or in combination with other components. Apart from food and packaging materials, food contact surfaces are also coated with antimicrobial agents to prevent microbial contamination. For instance, titanium coating on a stainless-steel surface was found to reduce the retention of *E. coli* and meat extract [34].

The term “nanocoating” is defined based on two perspectives. The first involves coating a surface with ultrathin layers of 10–100 nm thickness [35]; the second refers to coating a substrate with nanostructured materials [36]. In this review, the term “nanocoating” is considered to include both perspectives. Nanocoating differs from nanocomposites in such a way that in nanocomposites, the nanomaterials are reinforced/filled inside the matrix materials; while in nanocoating, the surface of the material is coated with nanomaterials or with any other materials in nano-sized thickness. In other words, nanocoating is an added layer, while nanocomposite forms the packaging material per se. Nanocomposites are often coated on the packaging materials, to provide synergistic effect of the reinforcement as well as the matrix material. For instance, coating a recycled carton sheet with a silver nanoparticle-based polystyrene coating was found to exhibit superior mechanical and moisture barrier properties, in addition to the antimicrobial activity of the silver nanoparticles [37]. Similar results were obtained for a paper sheet (obtained from rice straw) coated with polystyrene nanocomposites containing titanium dioxide nanoparticles doped or undoped with silver nanoparticles [38].

Nanocoatings with metal nanomaterials have been explored and adopted in various fields like automobiles, construction, electronics and communication, owing to their anti-fogging, self-cleaning, anti-microbial and de-polluting capabilities [39]. In addition, biopolymer-based nano-hybrid coatings incorporating metal oxides such as zinc oxide (ZnO) and titanium dioxide (TiO_2_) have also been investigated for corrosion protection of carbon steel, demonstrating improved barrier performance and enhanced corrosion resistance compared to conventional organic coatings [40]. Biopolymer-based nanocomposite coatings have also been demonstrated for applications like prevention of marine biofouling. In brief, biofouling is one of the major problems faced by aquatic industries, owing to the adsorption of organic molecules like proteins and polysaccharides, micro-organisms including bacteria, microalgae and diatoms and macro-organisms like barnacles, macro-algae and bivalves on wet surfaces. This can be prevented by chitosan-ZnO nanocomposite coating, which has been tested and proved for anti-diatom activity against *Navicula* sp. and antibacterial activity against *Pseudoalteromonas nigrifaciens*, a marine bacterium [41]. Layer-by-layer assembly of chitosan and rice husk ash nanosilica material-based coatings were found to possess anti-frosting, anti-fogging and anti-fouling properties [42]. Apart from these applications, nanocoating is also used in multifunctional textile treatments. For instance, a lignin-based multilayer nanocoating composed of magnesium lignosulfonate, chitosan, and monoammonium phosphate has been shown to impart self-extinguishing flame retardancy, as well as enhanced UV and antimicrobial protection to cotton fabrics [43].

Surface functionalization using nanocoating helps enhance the material properties such as barrier, antimicrobial, mechanical, self-healing, flame retardant, superhydrophobic, superhydrophilic, anti-reflective, and easy to empty characteristics [44,45]. Nanocoating applications in the food sector not only includes packaging or edible coatings, but also a wide range of applications like delivery systems for bioactive components and sensors for the detection of pathogenic microorganisms, contaminants and handling environment. Nanocoating can be performed on the inner or outer side of the surface or can be sandwiched as a layer [46]. The properties of nanocoatings depend on various factors like composition of coating, size of materials used, coating thickness, compatibility with the coating surface, technique adopted, etc. [47]. Hence, all these factors must be considered to optimize the coating and obtain maximum possible function. The composition of the coating and the size of the material primarily depends on the end-use of the coated material and the purpose for which coating is performed. Coating thickness is influenced by the coating technique adopted, characteristics of the coating solution—like viscosity, affinity, etc.—and the surface characteristics of the coated surface.

Techniques adopted for nanocoating include the sol–gel process, hydrothermal method/synthesis, chemical vapor deposition, electrodeposition, rotating coating, spray process, electrospinning, electrospray process, solution immersion process, and self-assembling methods [48]. As far as food packaging is concerned, techniques like atomic layer deposition, magnetron sputtering, electron beam evaporation and sol–gel method generally aid in preparation of coatings with better barrier properties. Thin layer coatings of aluminum oxide on paper board at various thicknesses such as 25, 50 and 100 nm were performed using these techniques to evaluate the effect of techniques on the gas barrier properties of the coatings. The results revealed that the atomic layer deposition technique, followed by magnetron sputtering were able to produce dense coating layers, that can reduce the gas permeability values to the best possible levels. Moreover, atomic layer deposited coatings were pin-hole free and dense, so that even nanoscale thickness can provide efficient gas barrier property [49]. Table 1 compares the key performance factors of various nanocoating fabrication techniques for biopolymer-based food applications.

Nevertheless, nanocoatings in the food sector are not explored completely, owing to the requirement of food grade, non-toxic, biocompatible, readily available and economical materials. Aluminum nanocoating on plastic materials, and organosilanes nanocoating on materials like glass bottles and jars can be used to enhance the performance of these materials [45]. Several researchers have coated polymers with nanomaterials to enhance the properties of the polymers. Among these, most of the studies for packaging materials include coating with silver, titanium dioxide, copper, gold, selenium, and ZnO nanomaterials to enhance the antimicrobial properties of the packaging material [58]. In few other cases, these nanomaterials are incorporated into some of the biopolymers like chitosan, carrageenan, alginate to enhance other properties like barrier and mechanical properties. The nanocoated packaging materials are preferred over multilayer packaging, owing to the lower material requirement and simpler process [36]. Thus, adopting nanocoating instead of multilayer films can be advantageous economically and ecologically. This article explores the applications of biopolymers in the nanocoating of food materials, food contact surfaces, and packaging materials.

## 2. Inorganic Materials in Nanocoating Applications

Inorganic materials including metal nanoparticles (such as silver and gold) and metallic oxide nanoparticles (such as TiO_2_, magnesium oxide (MgO), ZnO and aluminum oxide (Al_2_O_3_)) have been explored for nanocoating applications to enhance the mechanical, optical, magnetic, chemical, thermal and electronic properties and to generate antimicrobial, flame retardant, anti-corrosive, anti-fouling, anti-scratching surfaces, in the last few decades [59]. Moreover, the research focuses on improving these properties without affecting the intrinsic properties of the bulk material. However, hazardous effects of these nanomaterials on ecosystem are still not completely understood.

Silver in its nanoform is the most widely used nano-engineered antimicrobial material for applications in consumer goods. Nano-silver is coated on the surfaces of knives, food packaging, refrigerators, areas with a high possibility of microbial contamination like cutting boards, and meat processing equipment, to prevent biofilm formation and maintain an aseptic environment [60]. Silver-based organo-modified montmorillonite layered silicate was found to possess antimicrobial activity against *Salmonella* spp. [61]; while silver nanodots were anti-microbially active against *Pseudomonas fluorescens* and *Staphylococcus aureus* [62]. Several studies have proven that multilayer assembly aids in controlling and/or prolonging the antimicrobial activity of silver nanoparticles. Meng et al. [63] demonstrated that in situ synthesis of silver nanoparticles on silk matrix containing layer-by-layer assembly of poly acrylic acid and poly dimethyldiallylammonium chloride showed sustained antimicrobial activity of silver nanoparticles from modified silk for 120 h. Similarly, layer-by-layer deposition of polyethyleneimine and poly acrylic acid with silver nanoparticles on UV/ozone treated low-density polyethylene (LDPE) showed antimicrobial activity against *S. aureus* and *P. fluorescens* [64].

Photocatalytic surface disinfection of TiO_2_ aids in the peroxidation of phospholipids present in the cell membrane of microorganisms [65]. TiO_2_ nanocoating on LDPE films was found to exhibit bactericidal activity against *E. coli*. Evaluation of change in antimicrobial activity with respect to the light types like fluorescent and ultraviolet (UV) and length of exposure of light (1–3 days) found that the antimicrobial activity was increased with exposure to UV light, followed by fluorescent light. Moreover, exposure time also had a direct relationship with the antimicrobial activity [66]. TiO_2_ nanocoating can also be used for photodegradation of ethylene. On evaluating the photodecomposition of ethylene present in the head space of package containing tomato fruit packed with TiO_2_-coated oriented-polypropylene film, it was found that 88 ± 6% and 76 ± 10% of ethylene reduction was achieved using black light lamps and fluorescent light lamps, respectively [67]. These illustrations suggest that TiO_2_-nanocoated packaging materials can enhance the safety, quality and shelf life of food materials.

Similar to TiO_2_, ZnO in its nanoform is also known for its photocatalytic and photo-oxidizing effect on chemical and biological species. Reactive oxygen species generation is responsible for mitochondrial weakness, intracellular outflow and release in gene expression of oxidative stress, leading to cell growth inhibition and cell death [68]. The antibacterial activity of ZnO coated on LDPE was comparatively superior to those of TiO_2_, investigated in terms of inhibiting the growth of *E. coli* on fresh calf minced meat [69]. ZnO deposition on commercial polymers, can improve the barrier properties of the polymers, in addition to enhancing the antimicrobial properties [70]. Kayaci et al. [71] prepared electrospun polymeric nanofibers on which atomic layer deposition was used to form a coating with a layer of ZnO thin film with an approximate thickness of 27 nm or surface decorated with ZnO nanoparticles. The developed nanofibrous membranes were found to exhibit photocatalytic decomposition of Rhodamine-B, with a surface-decorated membrane showing the highest activity, owing to the high surface area. Moreover, ZnO nanocoating of 70 nm thickness was found to enhance the electromechanical behavior of cellulose film [72]. On the other hand, Kachoei et al. [73] demonstrated the ability of ZnO nanocoating to reduce frictional force and provide antibacterial activity against Streptococcus mutans on nickel-titanium wires. Also, ZnO-nanocoated bamboo timber was found to exhibit resistance against *Aspergillus niger* V. Tiegh and *Penicillium citrinum* Thom [74].

Silicon dioxide (SiO_2_), being one of the permitted food additives, has also been explored for application as a free flowing agent in powdered soups, clarifying agent in beers and wines and as a coating for food packaging materials and other food contact surfaces [75]. Moreover, concern still exists regarding the use of silica as a food additive, owing to the possibility of the presence of nano-sized materials in conventional form as a result of variation in natural size [76]. Nanoscale SiO_2_ for coating applications is prepared from various sources like silanes, organosilicates, tetraethylorthosilanes and chlorosilanes [77]. Moreover, silica-based nanocoating can enhance the alcohol resistivity of the rubber surface as well [78]. Silica-based nanocoating involving a mixture of tetraethoxysilane and 3-aminopropyltriethoxysilane in the presence or absence of incorporated layered double hydroxides were found to enhance the moisture and gas barrier properties of paperboard [79]. A similar study involving development of a superhydrophobic coating made of varnish modified with silica nanoparticles and polydimethylsiloxane silicone oil found that the coating had waterproof, easy cleaning and anti-frost characteristics. The researchers claimed that these coatings on paperboard can enhance its water resistance property [80]. Nano-SiO_2_ can also be used as a non-stick coating for food contact surfaces.

One of the innovative applications of inorganic nanoparticles is the creation of superhydrophobic or superhydrophilic surfaces, inspired by natural wonders like lotuses and elephant ears. It is nothing but the perfect combination of low surface energy, surface roughness and micro- and nano-sized particles. Though various inorganic nanoparticles like TiO_2_, Al_2_O_3_, and gold can generate superhydrophobic surfaces, SiO_2_ is preferred for its superior compatibility and ease of synthesis. These superhydrophilic and superhydrophobic surfaces are used for self-cleaning applications, such that the former involves the removal of dirt by the formation of a continuous film of water, while the latter involves the collection of dust particles through rolling droplets [44]. Han et al. [81] prepared stearic acid modified ZnO nanocoating with superhydrophobic characteristics and green photoluminescence properties for self-cleaning applications. Teisala et al. [82] used high-speed video system images to demonstrate the bouncing of water droplets falling on the surface of paperboard that consisted of a TiO_2_-based superhydrophobic coating.

These nanomaterials can also be advantageous in food applications, especially in packaging applications with the use of a barrier layer between the coating and the food material. Yet, the lack of studies on the toxicology of these materials and human studies regarding their impact remains a concern. The European Union has approved the use of nanomaterials like SiO_2_, ZnO, Titanium nitride (TiN) for use in plastic-based food contact applications for enhancing the mechanical, thermal and optical properties of materials [83]. Yet, these materials are not approved for antimicrobial applications [84].

While inorganic nanomaterials offer potent antimicrobial and barrier-enhancing properties, their practical application in food packaging is constrained by safety, regulatory, and performance limitations under real storage conditions. Silver nanoparticles provide broad-spectrum antimicrobial activity but raise concerns about cytotoxicity and ion leaching into food [85]. TiO_2_ and ZnO offer photocatalytic activity but require UV activation, limiting efficacy under typical storage conditions [68,86]. SiO_2_ excels in moisture barrier applications and superhydrophobic surface design but lacks inherent antimicrobial function [87]. Crucially, concerns over nanoparticle migration, cytotoxicity, and regulatory uncertainty continue to limit the real-world applicability of these inorganic materials in food packaging.

## 3. Organic Materials in Nanocoating Applications

Organic nanomaterials such as polysaccharide-based nanomaterials (cellulose, chitosan, starch), protein-based nanostructures (gelatin, zein, soy protein) and lipid-based nanomaterials have been widely explored for nanocoating applications because of their biodegradability, biocompatibility and excellent film-forming ability. These materials are particularly attractive for food, pharmaceutical and biomedical coatings as they are derived from renewable resources and are generally regarded as safe. Among the several other organic nanomaterials, cellulose-based nanomaterials are mostly preferred for their strength, structural stability and barrier properties [88]. Moreover, cellulosic nanomaterials are preferred over their macroscopic pulp fibers owing to the superior mechanical properties obtained as a result of the orientation of the crystalline region of the glucan chains along the fibril axis [89]. Cellulosic nanomaterials can be used for other applications including reinforcement in nanocomposites, self-standing thin films, and cushioning packaging materials. Nevertheless, in some cases nano-cellulose reinforcements in nanocomposites have a tendency to aggregate and, thus, result in poor dispersion leading to reduced performance [90].

Cellulose nanocrystals (CNC)/nanocrystalline cellulose (NCC), cellulose nanofibrils (CNF)/nanofibrillated cellulose/microfibrillated cellulose (MFC), electrospun cellulose nanofibers (ECNF), and bacterial cellulose (BC) are the most common cellulosic nanomaterials. Among these, the former two kinds are obtained by disintegrating macromaterials into nanomaterials (top-down approach) and have gained popularity in food packaging applications [91]. Moreover, CNF have gained popularity due to their exceptional physicomechanical properties including but not limited to oxygen barrier properties, low density, ability for chemical modification and high tensile properties [92]. Recent studies have further demonstrated that CNF-based nanocomposites can exhibit significant intrinsic antimicrobial activity against Gram-negative and Gram-positive bacteria, attributed to surface charge interactions and contact-active mechanisms rather than the release of external antimicrobial agents [93]. Depending on the shape, cellulosic nanomaterials can be nanorods, nanowhiskers, nanocrystals, nanofibers or nanofibrils [92]. These nanomaterials have potential applications in other fields like drug and pharmaceutical industries.

Hossain et al. [94] prepared nanocellulose-based nanocoating from biomaterial obtained from corn leaf biomass and evaluated the application of nanocoating for drug and capsule coating. The researchers presented the data on water absorption, odor testing, coating dye test, tensile test, pH, cellulose determination and chemical element determination and proved that the values were under the standards set by the American standard for testing and materials (ASTM). Cellulose nanomaterials are also used as reinforcements for edible coating applications. Cellulose nanocrystal (5 wt%) reinforced chitosan edible coatings were found to enhance the storage period of Bartlett (*Pyrus communis* L.) pears owing to superior gas barrier property [95].

The cellulose nanomaterials behave differently depending on the process involved in preparation, source, and type of coating method adopted. Various chemical methods adopted for extraction of cellulosic nanomaterials include acid hydrolysis, mechanical defibrillation, organosolv, dilute acid and 2,2,6,6-tetramethylpiperidine-1-oxyl (TEMPO)-mediated oxidation. Among these, TEMPO-mediated oxidation-based extraction is adopted widely owing to its ability to produce CNF with a high aspect ratio [96]. Rampazzo et al. [97] prepared CNCs from cotton linters (CL) and kraft pulp (KP), using the ammonium persulfate process and compared the characteristics of the CNCs. Additionally, the researchers analyzed the effect of nanocoating of the prepared CNCs on the polyethylene terephthalate films (PET), in terms of water resistance, optical properties, gas permeability, contact angle and coefficient of friction. KP-CNCs showed higher viscosity with shear thinning behavior; CL-CNCs suspension exhibited lower viscosity, such that it was not able to be detected using the cone-plate geometry which is used for measuring the viscosity. Due to this difference, when coating the PET substrate, CL-CNCs were applied at 8% (*w*/*v*) concentration in a single step; whereas, KP-CNCs were applied at 4% (*w*/*v*) in two steps, maintaining a similar final thickness. Both CNCs showed similar conductivity values. The coefficient of friction values of KP-CNC-coated PET was significantly higher than that of the CL-CNC-coated substrate. Nevertheless, both the dynamic and static values of coefficient of friction of CNC-coated PET were lower than the PET substrate alone. In addition, the study concluded that KP-CNC can be used as coating materials for PET as it provides better oxygen and carbon dioxide barrier properties and was little affected by temperature, at 0% RH.

Lavoine et al. [98] studied the characteristics of MFC-coated papers. The focus of the study was to compare papers coated by two different coating processes—bar coating and size press. Here, bar coating aids in coating only one side of the substrate; while size press coats both sides of the substrate at the same time. On the other hand, size press executes more pressure compared to that of bar coating. The 5 MFC coatings on the paper substrate using bar coating produced a coat weight of 7 g m^−2^ (approx.), whereas in the case of size press, the value was only 3 g m^−2^. This may be due to the movement of MFC into the paper due to the pressure applied, thus reducing the coat weight. However, Cobb index values were similar for both bar-coated and size-pressed samples. The increase in Young’s modulus was significantly higher in the case of the bar-coated samples than those of the size press-coated samples. On the other hand, the elongation at break in cross-direction increased for size press-coated samples, while it decreased for bar-coated samples. The study demonstrates the difficulty of uniform coating of MFC on a paper substrate. The coating was not homogeneous with one layer of coating. Moreover, this study aids in understanding the importance of coating method on the final properties of substrate.

Chitosan nanocoating helps to enhance the antimicrobial properties of the packaging material and the shelf life of the food materials. Nanocoating of clove and argan oil encapsulated into chitosan nanoparticles and nanofibers (low and high molecular weight chitosan, respectively) was carried out on poly lactic acid (PLA) substrate by means of coaxial electrospinning. The antioxidant and antimicrobial properties of the oil encapsulated coating were better than those of simple chitosan coating. These properties were, in turn, significantly superior than those of PLA substrate [99]. On coating apple slices with chitosan nanoparticles of different sizes like 110 nm and 300 nm and comparing with conventional chitosan coating (dipping), chitosan nanoparticle coatings were able to decrease the degree of browning of apple slices. Moreover, with the decrease in particle size of chitosan, the antimicrobial activity was found to improve, with chitosan nanoparticles of size 110 nm showing higher antimicrobial activity [100]. In a similar manner, chitosan coating in combination with nano-chitosan has been shown to exhibit a superior effect on browning and lignification retardation of minimally processed Zizania latifolia, compared to that of chitosan coating alone [101]. Comparing the two major organic coating systems, cellulose-based nanomaterials generally outperform chitosan in gas barrier and mechanical reinforcement due to their higher crystallinity, while chitosan’s cationic charge provides superior inherent antimicrobial activity, suggesting that hybrid systems combining both may offer the most balanced performance for food applications. Table 2 compares the key performance metrics for inorganic and biopolymer-based nanocoatings.

Beyond polysaccharide and protein systems, coatings inspired by biological membranes represent an emerging frontier in nanocoating design. Lipid bilayer-inspired coatings replicate the selective permeability of cell membranes, offering inherently low permeability to polar molecules while remaining responsive to environmental stimuli [109,110]. Phospholipid-based coatings deposited on solid substrates via vesicle fusion or Langmuir–Blodgett techniques have demonstrated excellent biocompatibility and anti-fouling properties on food-contact surfaces, largely due to their ability to resist non-specific protein adsorption, a primary step in biofilm formation [111]. Similarly, liposome-based nanocoatings can encapsulate lipophilic antimicrobial agents, enabling their stable incorporation into aqueous coating formulations and providing controlled, sustained release at the food surface [112]. The amphiphilic nature of phospholipids also facilitates their interaction with both hydrophilic biopolymer matrices and hydrophobic food surfaces, making them attractive as compatibilizing interlayers in multilayer nanocoating architectures. Although commercial adoption remains constrained by cost and processing challenges, biomimetic coating strategies present a biologically relevant alternative to purely synthetic systems and warrant further investigation for food-grade applications.

## 4. Multilayer Nanocoating

To provide a synergistic effect on the surface of the substrate, a combination of materials is preferred for nanocoating applications. The materials combined can be organic-inorganic, organic-organic or inorganic-inorganic. In general, compared to single layer nanocoating, multilayer nanocoating has proven to impart better properties to the coated material [113]. Most of the multilayer nanocoatings are performed using a popular technique called layer-by-layer (LbL) assembly. The LbL involves deposition/coating of cationic and anionic polyelectrolytes alternately to produce multilayers with nano-sized precision, that are organized through interactions such as electrostatic, acid-base or coordination interactions, hydrogen bonds and covalent bonds [114]. Moreover, LbL does not require the use of any sophisticated equipment and can be applied to substrates of any shape, size and material characteristics, as opposed to other high-cost deposition techniques that are only applicable to flat substrates [115,116]. Another advantage of LbL is the likelihood of the incorporation of active molecules during assembling of layers [117]. The multilayer coating of κ-carrageenan and quercetin loaded lecithin/chitosan nanoparticles developed by Souza et al. [114] can be referred to as an illustration.

The functional performance of LbL coatings is governed by several interacting parameters at the molecular level. The number of layers deposited directly determines the overall coating thickness and, consequently, barrier effectiveness, with each additional layer closing residual pinholes and extending the diffusion path for gas or moisture permeants. The pairing of oppositely charged polyelectrolytes (e.g., cationic chitosan with anionic alginate or κ-carrageenan) drives interlayer complexation through electrostatic interactions, producing a dense, coherent film that is more compact than either component alone. Deposition pH critically modulates this process: at pH values close to the pKa of the weak polyelectrolyte, partial ionization reduces charge density, yielding thicker, more diffuse layers with higher surface roughness, while deposition at pH values that maximize ionization produces thinner, denser, and smoother layers with superior gas barrier properties [118,119]. The incorporation of active agents at specific positions allows spatial control over their release profile, with agents deposited deeper within the multilayer stack exhibiting slower, more sustained release compared to those in outermost layers [120]. Collectively, these parameters provide a rational design framework for tuning LbL nanocoating architecture to specific food preservation requirements.

The antioxidant and antimicrobial properties of cellulose fibers were found to improve with the layer-by-layer deposition of chitosan and lignosulfonates [121]. Laufer et al. [119] prepared thin nanobrick walls of chitosan and MMT clay by means of LbL assembly and evaluated oxygen barrier and flame-retardant characteristics on PLA and polyurethane foam substrates, respectively. Here, the chitosan–clay nanobrick wall symbolizes MMT bricks held together by chitosan mortar. The results revealed that the 30 bilayer chitosan–MMT nanocoating with a 100 nm thickness on the PLA films showed enhanced oxygen barrier properties by 4 orders of magnitude. Similarly, the coating helped in reducing the flammability of polyurethane foam. This is evident from the 52% reduced peak heat release rate from 10 bilayer chitosan–MMT nanocoated (30 nm thickness) polyurethane foam than that of the uncoated foam samples. Several research works have demonstrated the application of LbL assembly technique with appropriate materials for enhancing the flame-retardant characteristics [122,123,124,125,126].

Medeiros et al. [127] prepared a nanomultilayer coating made of organic materials, namely, pectin and chitosan. The coatings were performed on PET layer and mangoes and the parameters like moisture and gas barrier properties were evaluated for nanomultilayer systems on PET substrate; while for multilayer nanocoating on ‘Tommy Atkins’ mangoes, the physico-chemical properties like mass loss, total soluble solids and titratable acidity were evaluated. The water vapor, oxygen and carbon dioxide permeability values of 5 nanomultilayers-coated PET were 0.019 ± 0.005 × 10^−11^, 0.069 ± 0.066 × 10^−14^ and 44.8 ± 32 × 10^−14^ g m/(Pa s m^2^), respectively; whereas, the values for pure PET were 1.42 ± 0.39 × 10^−11^, 2.5 ± 0.03 × 10^−14^ and 39.7 ± 22.9 × 10^−14^ g m/(Pa s m^2^), respectively. These values were either higher or similar to those of the nanomultilayer-coated PET. The multilayer nanocoating on mangoes revealed that after 45 days of storage, significant differences were observed between nanocoated and uncoated mangoes for all the analyzed parameters. A wrinkled and damaged appearance and microbiological spoilage were evident in uncoated mangoes, while these changes were not detected in nanocoated mangoes on 45th day of storage. A similar study was carried out with multilayer nanocoatings of alginate and lysozyme on ‘Coalho’ cheese and it was found that after 20 days of storage, the nanocoated cheese samples showed superior quality in terms of reduced mass loss, pH, lipid peroxidation, and microorganisms proliferation as well as higher titratable acidity, compared to those of uncoated cheese samples [128]. Likewise, multilayer nanocoatings of κ-carrageenan and chitosan on PET substrate were performed. The study involved the incorporation of methylene blue, a model cationic compound at different positions of multi-layered nanocoating and evaluation of its loading and release behavior. The researchers identified that as the distance from the first layer increases, the amount of methylene blue loaded also increases. This signifies the diffusion behavior of the methylene blue into multi-layered nanocoating, that helps in understanding the release of bioactive compounds for food applications [120].

For edible coating applications, LbL assembly of multilayer polysaccharides is performed on the surface of food materials. Martinon et al. [129] showed that multilayered coating of edible materials including trans-cinnamaldehyde, chitosan and pectin extended the shelf life of minimally processed cantaloupes. Similarly, layer-by-layer electrostatic deposition of chitosan and alginate was found to have a positive impact on shelf life of fresh-cut melon [130]. In another study conducted by Brasil et al. [131], it has been shown that incorporation of microencapsulated β-cyclodextrin and trans-cinnamaldehyde into multi-layered edible coating of chitosan and pectin extended the shelf life of minimally processed papaya. Alginate–chitosan multilayer nanocoating was found to enhance the microbiological and the physicochemical qualities of minimally processed mangoes. Briefly, the nanocoating extended the shelf life of fresh-cut mangoes up to 8 days at 8 °C, while uncoated fresh-cut mangoes were microbiologically safe for not more than 2 days [132]. Similarly, κ-carrageenan and lysozyme nanomultilayer coatings were found to reduce moisture loss and extend the shelf life of ‘Rocha’ (*Pyrus communis* L.) fresh-cut pears as well as whole pears. Moreover, coated pears exhibited superior quality parameters than that of uncoated pear samples [133].

## 5. Applications of Nanocoating in Food Sector

### 5.1. Nanocoating of Food Materials

Food materials are directly coated with edible coating materials to enhance their quality as well as the shelf life, including but not limited to whole and minimally processed fruits and vegetables, nuts, cheese, meat and seeds [134]. In this context, Chakraborty et al. [135] highlighted nanocomposite-antimicrobial systems for fish products; Poonia and Mishra [136], Das et al. [137], and Sharma et al. [138] summarized recent advances in edible nanocoatings; and Wang et al. [139] emphasized the significance of essential oil nanoemulsions and biopolymer-based edible coatings in food preservation. Furthermore, Travicic et al. [140] reviewed plant-based nanoemulsion edible coatings, with a particular focus on patent literature. Collectively, these articles highlight the increasing scientific and industrial interest in nanostructured edible coatings as promising approaches for improving food preservation and extending shelf life. The effect of nanocoating on selected fruits and vegetables are summarized in Table 3.

The nanocoating on food can be coated with edible material alone or with a combination of any of the active food grade ingredients. Candelilla wax-based nanocoating with tarbush extract has been proven to delay the senescence of Fuji apples as well as increase the shelf life of Golden delicious apples [153,154]. Likewise, calcium-alginate loaded with a silver-montmorillonite nanoparticles coating helped increase the shelf life of fresh-cut carrots by up to more than two months with proper packaging and storage [155]. In a similar way, cellulose whiskers reinforced alginate-acerola puree and montmorillonite reinforced alginate-acerola puree coatings have proven to reduce fruit weight loss, ripening rates and decay incidence as well as enhance ascorbic acid retention of acerola fruits. Comparatively, montmorillonite reinforced that alginate-acerola puree coatings were highly effective with superior color and visual acceptance [156]. Similarly, an alginate-nano-silver coating has proven to extend the shelf life of shiitake mushroom (*Lentinus edodes*), besides enhancing its preservation quality [157].

Edible nanocoating is also explored for a synergistic effect with other thermal and non-thermal treatments as well as packaging techniques and/or materials. The combination of pulsed light treatment and an antimicrobial coating made of modified chitosan consisting of carvacrol nanoemulsions was found to have an antimicrobial synergistic effect on the surface of minimally processed cucumber slices inoculated with *E. coli* [158]. Similarly, Severino et al. [159] evaluated the effects of γ-irradiation, UV-C and ozonated water treatments in combination with a coating made of modified chitosan consisting of 0.05% nanoemulsion of mandarin essential oil on antimicrobial activity against *Listeria innocua*, color and texture of green bean samples. The study concluded that γ-irradiation and UV-C treatments in combination with modified chitosan coating can have synergistic effect on antimicrobial property, without having a significant effect on physical properties. On the other hand, when evaluating the radiosensitization of *E. coli* and *Salmonella typhimurium*, it can be seen that the modified chitosan coating containing carvacrol nanoemulsion with modified atmospheric packaging provided a synergistic effect and exhibited higher radiosensitization than that without modified atmospheric packaging for both the tested organisms. Moreover, combined treatments of coating, gamma irradiation and modified atmospheric packaging provided better antimicrobial activity against *E. coli* and *S. typhimurium* on green beans for a storage period of 13 days at 4 °C [160].

Comparing the individual and combined treatments of nano-ZnO coating and ultrasound treatment on the quality parameters of fresh-cut kiwifruit for a period of 10 days proved that combined treatment can provide a synergistic effect and can extend the shelf-life of minimally processed kiwifruit, by lowering the ethylene as well as carbon dioxide production and water loss [161]. Likewise, a coating that consists of a silver-montmorillonite nanoparticle dispersed in sodium alginic acid solution combined with modified-atmosphere packaging was found to increase the shelf life of Fiordilatte cheese to more than 5 days, compared to that of 3 days with traditional packaging [162]. A similar study conducted by Mastromatteo et al. [163] showed that the combination of silver nanoparticles incorporated alginate coating with modified atmospheric packaging and brine as a covering solution were able to enhance the shelf life to 10 days.

Chitosan-based coatings have been explored more in edible coating of foods owing to their GRAS status and antimicrobial characteristics [164]. Chitosan nanoparticle edible coating on tomatoes delayed the ripening of fruits compared to those without coating [165]. Mohammadi et al. [166] proved that an edible coating with *Zataria multiflora* essential oil and which incorporated a chitosan nanoparticle coating had a positive effect on the physico-chemical properties of cucumber—including firmness, respiration rate, DPPH-radical scavenging activity and reducing power. It also reduced the decay level compared to cucumbers with a pure chitosan nanoparticle coating, which in turn showed superior effects compared to uncoated cucumber samples for a storage period of 21 days at 10 °C with 90–95% RH. Moreover, a chitosan–clay nanocomposite coating showed better quality lemon samples compared to those of uncoated fruit samples [167]. Nanochitosan-based edible coating, not only extended the shelf life of the strawberry, but also preserved the bioactive components during storage [168]. Nanochitosan emulsion coating drastically reduced the weight loss, respiration rate, ethylene production and peroxide values of apples compared to that of uncoated apple samples [169]. Likewise, a chitosan nanoemulsion containing a smaller lemongrass oil droplets coating showed a superior effect on reduction in microbial deterioration and enhanced shelf life of grape berries, compared to that of a coating containing larger droplets of lemongrass oil [170]. A similar study conducted with sodium alginate as the coating material showed similar results [171].

Bakhy et al. [172] investigated the effect of various coatings including chitosan–thymol/tripolyphosphate nanoparticles, gelatin–coconut fiber/titanium dioxide nanoparticles, chitosan–methyl cellulose/silica nanoparticles, gelatin–chitosan/silver/ZnO nanoparticles (LbL assembly) and gelatin–anthocyanin/kafirin nanoparticles on the quality parameters of fruits like apples, red grapes, tomatoes and sweet green pepper and compared them with an edible coating with no nano-materials. The study found that gelatin–chitosan/silver/ZnO nanocoating was better in maintaining the quality of the fruits. The quality was maintained for 63, 56, 42 and 35 days for apples, red grapes, tomatoes and sweet green pepper samples coated with gelatin–chitosan/silver/ZnO, respectively.

Though most of the studies deal with nanocoating applications for improving and/or maintaining the quality and the shelf life of the food material, nanocoating with nutrients can add nutritive value to the food material as well. For instance, vitamin-E (α-Tocopherol) edible nano-coating with potassium sorbate and lemon juice as antimicrobial and antioxidant agents improved the nutritive value of the fruit and enhanced the shelf-life of the fresh-cut apples [173]. Similarly, a study evaluated the effect of nanocapsules, nanospheres and nanoemulsion of α-Tocopherol with and without xanthan gum on the browning of fresh-cut Red Delicious apples. The study concluded that though xanthan gum helped in the formation of the coating, it did not show a superior antibrowning effect as compared to the nanosystems. Nanocapsules were able to better prevent the browning of apple slices, followed by nanospheres. Moreover, these nanosystems helped reduce the loss of firmness in the apple slices [174].

### 5.2. Nanocoating of Packaging Materials

Packaging is one of the major areas of focus in the food sector, as packaging material plays a vital role in protecting the food material from the external environment. To avoid deterioration in sensory properties, loss in nutritional properties and the growth of harmful microorganisms, packaging material must perform appropriate functions in terms of protection against light, heat, moisture, gas and so on [47]. Hence, the development of innovative food packaging materials and enhancement of property of existing materials is of prime importance. Nanotechnology plays a vital role in this context. This section reviews the applications of nanocoating in enhancing the performance of packaging materials, emphasizing the biopolymer-based nanocoating.

The barrier performance of biopolymer nanocoating is governed by molecular-level mechanisms that merit consideration alongside empirical results. Gas and moisture transport through polymer coatings follows a solution-diffusion mechanism: permeants sorb into the coating at one interface, diffuse through the polymer matrix under a concentration gradient, and desorb at the other side. The diffusivity of a permeant is strongly influenced by the free volume within the polymer network, a parameter determined by chain flexibility, crystallinity, and cross-link density. Highly crystalline materials such as cellulose nanocrystals present a tortuous diffusion path to gas molecules, dramatically reducing permeability. In contrast, amorphous or hygroscopic polymers like alginate absorb moisture, which plasticizes the chain segments, increases free volume, and compromises barrier integrity at high relative humidity. Electrostatic interactions in layer-by-layer systems further restrict chain mobility at the bilayer interface, contributing to the superior barrier performance often observed in multilayer architectures compared to single-layer equivalents [175,176,177,178]. Understanding these structure–property relationships is essential for the rational design of durable nanocoatings. A related concept gaining traction in drug delivery that holds potential for nanocoating systems is hydrophobic ion pairing (HIP). HIP involves the formation of ion pairs between charged macromolecules (such as cationic chitosan or anionic alginate) and oppositely charged hydrophobic counterions, yielding complexes with reduced aqueous solubility, enhanced membrane permeation, and tunable release characteristics [179]. In the context of food nanocoatings, HIP could be leveraged to improve the loading efficiency and controlled release of hydrophilic antimicrobial or antioxidant agents into hydrophobic coating matrices, or to modulate the interaction between biopolymer coating layers and lipid-rich food surfaces. Although this application remains largely unexplored in food packaging, the mechanistic parallels with drug delivery make it a promising direction for future investigation.

#### 5.2.1. Coating of Paper-Based Packaging Materials

Though paper-based packaging materials are advantageous over polymeric packaging materials in terms of sustainability and cost, it is not adopted for wide commercial applications, owing to its poor water resistance characteristics [80]. However, the global paper-based packaging market is anticipated to expand at a cumulative annual growth rate of 2.3% during the period from 2024 to 2033 [180]. Recent advancements show the potential application of biopolymer-based coating for surface coating of paper-based materials to enhance the properties like gas barrier and mechanical properties, electrical conductivity, photocatalytic activity, antimicrobial activity, magnetic property, smoothness, moisture-repellent characteristics, transparency and opacity [181,182,183]. According to Kunam et al. [184], research interest in sustainable packaging materials and sustainable barrier coatings for paper has grown considerably in recent years. To enhance the required properties of paper, appropriate nanomaterials must be chosen. For instance, coating paper with materials like nano-ZnO and nano TiO_2_ enhances opacity by reducing the transmission of light by means of refraction, as well as improving the antimicrobial property; while iron (III) oxide nanocoating helps with the preparation of magnetic papers [185]. Furthermore, poly caprolactone (PCL) coatings modified with SiO_2_ and Al_2_O_3_ nanoparticles on paperboard decreased the moisture permeability and improved the mechanical properties of paperboard [186]. Likewise, halloysite nanotubes reinforced sodium caseinate coating on paper enhanced its mechanical, grease resistance and moisture barrier properties. The study found that the optimum coating weight and halloysite nanotube (HNT) content to obtain maximum favorable response on the tested parameters were 15.99 g/m^2^ and 1.45 wt% [187].

Cellulosic nanomaterials are used as coating material to enhance the barrier and mechanical properties of paper-based packaging materials. The coating containing nanofibrillated cellulose and 10% chitosan nanoparticles on paperboard, showed enhanced tensile and grease proof properties, and reduced porosity and water absorption. However, the coating had no significant effect on water vapor permeability. The researchers claimed that this is due to the porous nature of nanocellulose and the ability of chitosan nanoparticles to absorb and diffuse water vapor molecules, owing to the presence of amino and hydroxyl groups [92]. On the contrary, multilayers of microfibrillar cellulose and shellac coating on paperboard and papers had drastically influenced the oxygen and moisture barrier properties. The water vapor transmission rates decreased in such a way that is considered a high barrier in food packaging. However, the addition of microfibrillar cellulose does not enhance the barrier properties significantly, layers of shellac has helped cover the “pin holes” [188]. A similar study involving coating kraft paper (wrapping paper) and greaseproof paper with MFC have proved to enhance the gas barrier and oil barrier properties with reduced porosity [189]. Similarly, multilayer super-repellent biopapers were developed by Lafraya et al. [190], using electrohydrodynamic processing, where ultrathin fibers of PLA, PCL, or PHBV were electrospun onto cellulose paper followed by electrospraying of hydrophobic silica microparticles. The hierarchical micro/nanostructured coatings exhibited water contact angles above 155° and sliding angles below 10°, demonstrating excellent water vapor barrier performance and food-repellent behavior suitable for easy-emptying food packaging applications.

Bionanocomposite suspension coating made of wheat gluten, 7.5% cellulose nanocrystals and 0.6% TiO_2_ was performed on unbleached kraft paper sheets and properties like breaking length, burst index and antimicrobial properties were evaluated with different layers of coating (1, 2 and 3 layers). The results revealed that a maximum of 56% and 53% enhancement in breaking length and burst index were achieved for paper coated with 3 layers. And, antimicrobial activity of coated papers, investigated against *Saccharomyces cerevisiae*, *E. coli*, *S. aureus* showed that 98.5% reduction in surviving number was observed compared to that of uncoated papers [191].

#### 5.2.2. Coating of Polymeric Packaging Materials

Nanocoating with bio-based polymeric food packaging materials is of prime interest, especially for enhancing barrier, mechanical and antimicrobial properties [192]. Coating is also performed on the surface of few synthetic packaging materials to enhance various performances like antimicrobial, self-healing, fog-resistant/antifoaming properties [193,194,195]. For instance, coating of starch-coated polyethylene films with ZnO nanoparticles were found to exhibit biocidal action against *E. coli* [196]. Likewise, polyvinyl chloride (PVC) films coated with ZnO powder showed antimicrobial activity against *E. coli* and *S. aureus* [197]. Sadeghnejad et al. [198] found that silver nanoparticle coating on polyethylene films, surface modified by corona air plasma can act as a potential antimicrobial food packaging film with activity against *S. aureus* and *E. coli*. Application of nanocoating on polymeric food packaging materials is performed to enhance the properties of the packaging material, and hence the food packed in it.

Halász [199], carried out LbL assembly of chitosan and CNC on PLA film and bottle to enhance the barrier properties. The study found that a four bilayer coating of chitosan and CNC provided transparent barrier coating and showed a 29 and 26% reduction in moisture vapor permeability compared to those of neat PLA films and bottles. A similar study was carried out by coating multilayers of chitosan and sodium alginate on PET substrate with the outcome that the nanolayered coating had significant effect on hardness and thermal properties [200]. Several studies were performed by coating multi-layered nanostructures of edible biodegradable materials like κ- carrageenan, lysozyme, alginate and chitosan in various combinations to enhance the barrier and antimicrobial properties of PET substrate [128,133,201].

#### 5.2.3. Coating of Glass or Metal Containers

Nanocoating glass and metal containers is mainly focused on preventing physical damage as well as enhancing the material’s properties like mechanical, antimicrobial and thermal properties. While coating, the inherent properties of these materials, like optical properties and sealability, must not be affected. Though biopolymer-based nanocoating on these kind of containers has not been explored completely until now, some of the metal nanomaterials are coated on the surface of these materials. Rai et al. [202] coated poly ethyleneimine modified glass surfaces with cefaclor reduced spherical gold nanoparticles of size 52–22 nm and found that the glass surfaces with antimicrobial coatings were strongly effective against *E. coli*. Moreover, the glass surfaces were able to exhibit antimicrobial activity several times on repeated usage (more than 5 times) and the coatings were effective even under adverse conditions of pH 3 and 10. Another coating made of a halloysite-based polymer (polymethyl methacrylate and polystyrene) nanocomposites, synthesized using ultrasound-assisted solution blending method were evaluated on various types of soda-lime glasses including clear, bronze, green and gray. The study revealed that halloysite-based polymer nanocomposite coatings were able to enhance surface’s hydrophobic properties and the scratch resistant characteristics of the coated glass materials, without having an effect on the inherent spectral properties [203]. Makwana et al. [204] coated cinnamaldehyde nano liposomes encapsulated with polydiacetylene and N-hydroxysuccinimide bilayers on aminolysed PLA and glass surfaces. Their results found that glass surfaces immobilized with nanoliposomes showed antimicrobial activity against *E. coli* and *Bacillus cereus*, while nanoliposomes immobilized PLA surfaces did not exhibit any antimicrobial activity. The researchers claimed that aminolysis treatment led to less density of amine groups on the surface of PLA films, which further affected the immobilization of cinnamaldehyde liposomes, leading to insignificant antimicrobial activity. Jin and Gurtler [205] claimed and demonstrated that a PLA coating consisting of allyl isothiocyanate, nisin or a ZnO nanoparticles-coated glass jar can be used as effective antimicrobial packaging for liquid eggs, owing to its antimicrobial activity against three heat-resistant strains of *Salmonella* spp.

Nanocoating cans that are in contact with food is performed to prevent the corrosion of cans and to protect the food from spoilage and contamination upon contact with an uncoated metal surface. Polymeric coating with nanocarriers aids in serving this purpose [206]. Plasma nanocoating of aluminum cans and tubes aids in protecting the aluminum from corrosion [102]. Only limited studies are available on nanocoating metal cans used in food applications.

### 5.3. Nanocoating in Active Packaging Applications

Active packaging systems are packaging systems that perform actions such as antioxidant, antimicrobial or other biocatalytic functions with the aid of active agents present in them to enhance the quality, safety and/or the shelf life of the food packed in it [102]. According to Wilson et al. [207], edible coatings are considered to be a subset of active packaging. Moreover, coatings are better suited than films for active packaging applications, owing to the higher availability of active agents for migration in solution form [208]. On the other hand, the solution characteristics differ for coatings from those of films. For instance, coatings require high solubility; whereas, low solubility is preferred for films used for packaging [209]. Nanocoating plays a significant role in active packaging application, where nanomaterials aid in protection of the food materials by interacting either directly with the food or the environment [210]. The technique adopted for nanocoating materials with active ingredients can be migratory or non-migratory. Having said that, the former includes technologies that aid in the migration of the active ingredient to the packed food such as embedding, non-covalent immobilization and selected LbL deposition techniques; whereas, the latter includes technologies that result in the retention of the active ingredient in the packaging material itself such as covalent immobilization, photografting and some of the selected LbL deposition techniques [102].

Coatings of packaging materials with metal nanomaterials have enabled the development of diverse active packaging systems, evaluated for their impact on food shelf life, sensory and quality parameters. ZnO and TiO_2_-based antimicrobial food packaging systems are developed owing to their photocatalytic activity that leads to microbial cell death [210]. The antimicrobial property of these coated nanomaterials varies not only with the type of nanomaterial, but also with the properties of nanomaterials such as size, concentration, morphology and defects, UV illumination, presence of other additives, interaction between the substrate and the coating material, method of coating, and thickness of coating [68]. Moreover, these nanomaterials are advantageous over silver nanoparticles, especially for food packaging applications, owing to the lower toxicity of ZnO and non-ionization characteristics of TiO_2_.

Li et al. [211] developed an active packaging made of PVC coated with nano-ZnO powder and evaluated the effect on fresh-cut ‘Fuji’ apples at 4 °C for 12 days. Based on the observations like reduced fruit decay rate and accumulation of malondialdehyde, suppressed wound-induced ethylene production, decreased polyphenoloxidase and pyrogallol peroxidase activities and lower browning in apple samples packed nano-ZnO-coated PVC film, in comparison with uncoated PVC film packed samples, the study concluded that nano-ZnO-based active packaging can be suitable for enhancing the shelf-life of minimally processed ‘Fuji’ apples.

Chitosan is one of the widely used coating materials for active packaging, owing to its antimicrobial and film-forming characteristics [208]. Chitosan is combined with other antimicrobial agents like benzoic and sorbic acids in original as well as nano-form to provide enhanced antimicrobial activity [212]. The effect of ZnO-based chitosan nanocomposites-coated low density polyethylene film (LDPE) was studied on the quality parameters of okra Abelmoschus esculentus—such as, pH, total soluble solids, moisture content and weight loss. The study found that antimicrobial packaging material was able to reduce the microbial growth on the samples tested after 12 days of storage. Moreover, the nanocomposite-coated LDPE packaging material did not show any significant effect on the evaluated quality parameters of okra samples [213].

The applications of nanocoating of antioxidants to prevent oxidative degradation of the food products and biocatalytic nanocoatings like biocatalysts to break down undesirable/harmful products that are responsible for quality deterioration of food materials has not been completely explored. Though active packaging materials with incorporated antioxidants and biocatalysts exist, their application in nanocoating form can enhance the activity of the active ingredient. Very few studies have explored the use of nanostructure coating on the packaging materials to enhance their antioxidant property. For instance, Fabra et al. [214] coated electrospun nanostructures consisting of α-tocopherol, an antioxidant encapsulated with various matrices such as whey protein isolate, zein and soy protein on thermoplastic wheat gluten film substrate. The study showed that the nanostructure coating enhanced the moisture barrier property of wheat gluten films. Also, delayed α-tocopherol release and enhanced α-tocopherol stability to industrial sterilization process was observed for nanostructures made of zein. Consequently, this proof-of-concept study proposed the feasibility of use of the nanostructure-coated films for potential application in food contact applications for liquid products like milk and juice for improving health benefits, besides preventing lipid oxidation. On the other hand, Bharathi et al. [215] compared the ability of zein nanofiber-coated chitosan film to prevent browning of fresh-cut apples slices with that of uncoated chitosan and zein films and proved that zein nanofiber coating on chitosan film substrate can have promising application in preventing the browning of minimally processed fruits and vegetables, owing to the improved antioxidant activity of zein nanofibers obtained as a result of high surface to volume ratio.

### 5.4. Nanocoating of Food Contact Surfaces

Food processing equipment is nanocoated mainly to prevent the growth of microbes or to prevent fouling. Materials like aluminum are coated with aluminum nanoparticles to reduce surface roughness [216]. Anti-fouling coatings in the form of hydrophobic and/or oleophobic coatings are performed on heat exchangers in food processing plants. These kinds of coatings can reduce the time of cleaning in place (CIP) [217]. Kananeh et al. [218] investigated the anti-fouling characteristics of nano-composite coatings made of various polymer matrices like epoxy, polyurethane or polyamide that were cross-linked with per-fluorinated monomers or oligomers and ceramic reinforcement particles. The coated plates of gasketed heat exchanger were installed in the milk heating section of a pasteurizer and the results revealed that polyurethane-based coating had the thinnest layer deposit. Moreover, the coating reduced the time required for CIP by 70% compared to that of standard stainless-steel plates. In a similar manner, anti-fouling coating made of multiwalled carbon nanotubes dispersed in polytetrafluoroethylene on plate heat exchanger surfaces, reduced the risk of under-processed milk and reduced the energy consumption [219].

Food contact surfaces and processing equipment are a major source of microbial hazards. Hence, proper maintenance of these surfaces is mandatory. Nanocoating with antimicrobial substances aids in preventing the growth and contamination of microorganisms. In general, silver coatings are performed on selected dishwashers and refrigerators, owing to silver’s high toxicity towards most of the harmful microorganisms [220]. However, silver- and zinc- containing zeolite paints were able to inactivate only the vegetative cells of *Bacillus anthracis*, *B. cereus* and *B. subtilis*, but not their spores present on a stainless steel surface [221]. Similarly, TiO_2_ also showed strong antimicrobial activity against a wide range of microorganisms. Yemmireddy and Hung [222] evaluated the physical stability of TiO_2_ in combination with various binders such as shellac, polyurethane and polycrylic and found that polyurethane, as a binder, showed superior adhesion strength and scratch hardness; while polycrylic, as a binder, showed more physical stability and higher retention of antimicrobial property on repeated usage. The study also evaluated the effect of UV-A light intensity on the bactericidal property of TiO_2_ and found that the bactericidal property enhanced with an increase in UV-A light intensity. Superhydrophilic and superhydrophobic nanocomposite coating of titanium dioxide and carbon nanotubes-polytetrafluoroethylene, respectively, on stainless steel were found to reduce bacterial retention [223].

Most of the studies discussed above are based on metal nanomaterials; biopolymer-based nanocoating on the surface of food processing equipment is not explored, owing to the difficulties in processing and handling. Moreover, the durability of biopolymer-based nanocoating is comparatively inferior to that of metal nanomaterials. Few studies are available on the application of metal nanomaterials synthesized using chitosan as capping agent for an anti-fouling coating. Abiraman et al. [224,225] developed anti-fouling coatings made of chitosan decorated copper nanoparticles of size less than 2 nm and chitosan adorned ZnO nanorods of about 15 nm width and 110 nm length and demonstrated anti-fouling behavior against green algae such as Arthrospira and Chlorella and marine algae, named, Amphora. However, Bulwan et al. [226] proved that chitosan-based nanocoatings can be used as an anti-fouling, antibacterial and anticoagulant coating on surfaces like silicon and glass. And carboxymethylcellulose and chitosan-based antimicrobial multilayer films can be used as a coating material for clear overlay appliances, that are commonly used in orthodontic fields [227]. Similarly, future research can focus on biopolymer nanocoating on food contact surfaces, so that the possible hazard of metal nanomaterials migrating to food can be eliminated.

## 6. Interaction with the Food Materials

Whenever a novel material is explored for application in the food sector, its interaction with food materials is one of the obligatory things to be considered. Specifically, when nanomaterials are used in direct contact with food materials, their interaction with the food material is of prime concern due to the size of the material and modification in intrinsic property obtained as a result of high surface to volume ratio. Moreover, the behavior of the nanomaterials in biological systems can be different from those analyzed instrumentally. Interaction, in the broader sense of packaging, involves three major phenomena, namely migration, permeation and sorption [228]. Yet, while considering the toxicity of nanomaterials, the latter two are least taken into consideration. Moreover, determination/monitoring of the migration of nanomaterials into food is of vital importance to ensure the safety of the food. Hence, this section focuses mainly on the migration of nanomaterials into food systems.

A fundamental concern surrounding nanomaterial-based coatings is their regulatory standing. Although bulk forms of ZnO (21 CFR 182.8991) and TiO_2_ (21 CFR 73.575) are approved by the FDA [102], these approvals do not extend to their nanoscale counterparts, which exhibit distinct physicochemical properties, including higher surface area, increased reactivity, and enhanced biological interactions, necessitating independent safety assessments. Furthermore, although nano-ZnO has been considered relatively safer compared to other nanomaterials, evidence from multiple studies indicates that oral exposure may induce cytotoxic and inflammatory effects, highlighting the need for caution in its use in direct food-contact applications [229]. Notably, the European Union (EU) banned TiO_2_ as a food additive (E171) in 2022 citing genotoxicity concerns [230], while regulatory agencies in other regions, including the FDA, have not yet implemented a similar prohibition, reflecting the ongoing divergence in global regulatory positions on nanomaterial safety. Similarly, nano Ag is neither approved as a food additive nor classified as GRAS by the FDA, and the EU has not authorized its use in food contact applications either. A similar regulatory gap applies to many other nanoparticles, including nano Al_2_O_3_, nano CuO, and nano SiO_2_, where bulk-form approvals exist in certain jurisdictions but explicit authorization for nanoscale use in food contact materials is absent or pending. Beyond the EU and the United States, other major markets including Canada, China, India, and Brazil similarly lack nano-specific food packaging regulations, instead relying on existing general food safety legislation or case-by-case risk assessment approaches, further compounding the fragmented nature of the global regulatory landscape. This regulatory divergence across global agencies underscores the need for internationally harmonized frameworks governing nanomaterial use in food packaging.

Migration is the diffusion/transfer of any compounds that are present as a part of packaging material into the food system. Applications involving nanocoating the surfaces of packaging material that is not in direct contact with the food material or sandwiching the nanocoated layer between various layers of packaging material can possibly reduce migration. But nanocoating directly on food—such as edible coatings or in packaging materials or other food contact surfaces—is a serious concern. Most of the studies have reported that nanomaterials that migrated from nanocoating that was performed on packaging material was within a safe limit. A study involving assessment of silver nanoparticles migration from edible coating into sausages demonstrated that silver nanoparticle concentration on the surface can be reduced to as low as 5.3 nanograms of silver nanoparticle per gram of sausage by simple washing. Moreover, no considerable migration of silver nanoparticles to the interior of the sausage was detected [231]. Similarly, studies on nanosilver-coated polyethylene (PE) containers and nanocoated PE films (PEF) assessed using pasteurized orange juice (without pulp) at 70 °C for 10 days reported migration levels of 28.92 µg/L and 5.66 µg/L, respectively; under aqueous conditions (water, 40 °C, 6 weeks), PEF recorded a migration level of 6.4 µg/L, underscoring that the substrate type and food simulant conditions considerably influence the extent of nanosilver migration [232]. However, a study on Al_2_O_3_ nanocoating reported that Al_2_O_3_ migration to 3% acetic acid was as high as 0.9 mg/dm^2^, which led to migration of higher concentration (0.5 mg/dm^2^) of aluminum. Hence, the study suggests the use of an additional layer after nanocoating to reduce migration limit [233].

European Union sets limits/threshold level for migration of compounds from food contact surfaces to the food system depending on the toxicity and consumption rate; while according to FDA, materials used in direct contact with the food should be recognized as GRAS or must have been proven as safe [228]. According to European Commission, to assess the migration limits, simulants such as water, vegetable oil, acetic acid (3% *v*/*v*) and ethanol (10–50% *v*/*v*) can be used, depending on the type of food system in which the material comes in contact [84]. Since most of the studies carried out are under these simulated conditions, a complete assessment of nanocoating in food systems is lacking. When evaluating the migration of nanosilver from nanocoatings on the surface of polyester and LDPE, Hannon et al. [234] explained the difficulty involved in analytical techniques in differentiating silver nanoparticles from Ag+ in milk. Complete knowledge on nanomaterials toxicity and the requirement of advanced technologies and assessment method can help evaluate the interaction of nanomaterials present in nanocoating with the food material as a whole.

## 7. Perspectives

Despite notable progress, biopolymer-based nanocoatings for food applications are still at an early stage of development, with several emerging directions likely to shape future research. One promising area is the use of stimuli-responsive biopolymers that react to changes in pH, temperature, or enzymatic activity, enabling the targeted, on-demand release of antimicrobial or antioxidant agents at the onset of spoilage. Advances in digital manufacturing, such as inkjet printing and electrospray deposition, also offer new possibilities for applying precisely patterned coatings on complex food surfaces, potentially overcoming some of the scalability limitations associated with conventional dip- or spray-based methods.

In parallel, there is a growing need to evaluate the environmental impact of these systems more rigorously. Incorporating life cycle assessment (LCA) approaches, from raw material sourcing through to end-of-life disposal or compostability, will be important for validating sustainability claims and supporting regulatory approval. At the same time, the increasing use of computational tools, including molecular dynamics simulations and machine learning-based optimization, is expected to streamline formulation design and reduce reliance on trial-and-error experimentation.

Finally, the lack of harmonized international standards for testing and migration assessment of nanoscale coatings remains a major barrier to commercialization. Developing such standardized protocols will be essential to bridge the gap between laboratory-scale innovation and safe, widely accepted food packaging applications.

## 8. Conclusions

In recent years, the integration of coating technologies with nanotechnology has gained significant attention as a promising approach to developing advanced, functional surfaces for food-related applications. Some of the water-based coating such as NanoSeal^TM^ and Bairicade XT^TM^ involving clay platelets are adopted to enhance the gas barrier properties of traditional packaging including polyester, polypropylene, nylon and PLA involving indirect food contact commercial applications [235]. Apart from nanocoating, printing active ingredients is also explored to enhance the functionality of the packaging film [236].

A critical but underexplored dimension of nanocoating technology is the challenge of transitioning from laboratory-scale proof-of-concept to continuous industrial processing. Layer-by-layer assembly, while highly versatile at bench scale, requires multiple sequential deposition and rinsing steps that are difficult to execute at commercial line speeds without sacrificing coating uniformity or interlayer integrity. Similarly, electrospinning produces nanofiber coatings with excellent functional properties but suffers from low throughput and challenges in achieving uniform deposition on non-flat food surfaces such as whole fruits and vegetables. Atomic layer deposition delivers exceptionally dense, pinhole-free coatings but demands vacuum conditions and high capital investment incompatible with most food processing environments. Spray coating and dip coating are the most scalable approaches for biopolymer-based systems, but controlling coating thickness and uniformity across irregular substrates at industrial speeds remains technically demanding. Addressing these barriers will require investment in continuous coating equipment adapted for food-grade biopolymer dispersions, as well as inline quality control systems capable of verifying coating thickness and integrity in real time [102,115,237,238].

A key gap that remains largely overlooked in the current literature is the long-term stability of biopolymer nanocoatings under realistic supply-chain conditions. Most studies assess coating performance over relatively short periods, typically days to weeks, and under tightly controlled laboratory settings. These conditions do not adequately capture the temperature fluctuations, mechanical stresses, and extended storage durations encountered during actual food distribution. In particular, the hygroscopic nature of many biopolymers makes them prone to swelling, delamination, and a gradual loss of barrier properties when exposed to varying humidity levels. At present, there is no standardized approach to evaluate coating durability over a representative supply-chain timeline. Future studies should therefore incorporate more practical testing conditions, including simulated transport vibration, repeated temperature cycling across cold-chain ranges (−20 °C to ambient), longer storage durations (e.g., 3–6 months), and exposure to humidity levels typical of packaging environments (60–90% RH). Developing and adopting such protocols would help bridge the gap between laboratory findings and real-world applicability. Due to economic, performance and regulatory concerns, manufacturers are reluctant to adopt these nanocoatings for direct food contact applications. Other practical difficulties hindering the commercial application of nanocoating include lack of complete knowledge on the interaction between the substrate and the coating material, which directly impacts the durability and stability of the coating. Also, bulk coating of substrates like fruits and vegetables with different shape and size is difficult and time-consuming [239]. However, focusing research and development on durable nanocoating with a low cost and convenient method can boost the quality and shelf life of food products drastically. Also, regulations related to non-migratory applications and level of migration, testing methods and standard simulants for migratory applications, properties of materials used for nanocoating, their characterization techniques, impact of nanocoating on the bulk material properties and processing, tests regarding delamination of coatings during end-use and/or processing, level and nature of active ingredients used in coatings for food contact and non-contact applications must be taken into consideration by regulatory bodies worldwide or countrywide [102].

To set standards related to nanocoating and to bring it into practical application, a complete knowledge on the characteristics of the coating materials during processing, storage, and food contact and its effect on human health, environment, properties of food materials is important. Non-toxic, food grade biopolymer-based nanomaterials can be a better alternative to metal-based nanomaterials in food packaging application. Lack of information on these aspects opens the possibility of future research exploring the potential application of biopolymer-based nanocoating to enhance the quality, safety and shelf-life of food materials. This can directly or indirectly prevent loss of food products, their market value and the brand name of the manufacturers, besides enhancing the consumer acceptability and palatability of food. This can also be a boon to prevent diseases related to nutritional deficiencies by incorporating nutrients and their active substances as edible coatings.

## Figures and Tables

**Table 1:** Coating techniques and their suitability for biopolymer-based food nanocoating applications.

Technique	Typical Thickness	Uniformity	Scalability	Adhesion to Substrate	Biopolymer Suitability for Nanocoatings	Primary Limitation for Food Use
Dip coating	100 nm–several µm	Moderate	High	Good	High (Widely applied to fresh produce)	Drainage effects; Excess material waste on irregular surfaces
Spray coating	100 nm–10 µm	Moderate-high	High	Good	High (Fresh produce; Edible and active packaging coatings)	Thickness control variability, especially on curved surfaces
Layer-by-layer assembly	5–500 nm total (1–50 nm per layer)	Very high	Low-moderate	Excellent (Electrostatic/H-bonding)	High (Protein/polysaccharide multilayers; Responsive release systems)	Multi-step; Slow processing speed; Difficult to scale
Sol–gel coating	10–500 nm	High	Moderate	Good (Improves with crosslinking)	Moderate (Polysaccharide-inorganic hybrid systems; Silica-based barriers)	Requires solvents; Curing conditions limit direct food contact applications
Electrospinning	50 nm–5 µm (Fiber diameter; Mat thickness variable)	Moderate	Low	Moderate (Post treatment or additives needed)	High (Active/antimicrobial packaging; Encapsulated bioactive delivery)	Low throughput
Electrospraying	50–500 nm	High	Low-moderate	Good	High (Precise encapsulation and deposition of bioactives)	Solvent use/Scale-up complexity

References: [50,51,52,53,54,55,56,57].

**Table 2:** Comparative summary of key performance metrics for inorganic and biopolymer-based nanocoatings in food applications.

Parameter	Inorganic Nanocoatings	Biopolymer-Based Nanocoatings
Gas barrier	High-Very high	Moderate-High (humidity dependent)
Mechanical strength	High	Moderate
Moisture resistance	High	Low-Moderate (Mostly hygroscopic)
Antimicrobial activity	Variable	Moderate
Biodegradability	No	Yes
Toxicological profile	Concerns over nanoparticle migration	Generally favorable, some gaps remain
Cost	Moderate-High	Low-Moderate

References: [102,103,104,105,106,107,108].

**Table 3:** Effect of nanocoating on selected fruits and vegetables.

Food Material	Coating Material	Particle Size/Structure	Primary Microbes Tested	Storage Conditions	Storage Duration	Key Preservation Metrics	References
Banana	Chitosan, caraway essential oil	105 ± 8.4 nm	*E. coli* and *S. typhi*	(25 °C)	7 days	↓ weight loss; maintained firmness, color, TA; ↑ antioxidant enzymes	[141]
Banana	Alginate, Carrageenan, Pectin, ZnO NP	-	-	Room temperature	15 days	↓ Weight loss, pH, TSS; ↑ TA, reducing sugars, firmness	[142]
Banana	Cassava starch, xanthan gum, ZnO NP	-	*E. coli*, *S. aureus*, *F. oxysporum*	-	-	Antibacterial (1% ZnO); antifungal (2% ZnO); extended shelf life	[143]
Banana	Chitosan, cellulose nanofibers (garlic skin), nanocurcumin	Nanocomposite	*E. coli*	-	+8 days vs. control	↓ Weight loss/TSS; maintained TA; gas barrier effect	[144]
Banana	CS, gum Arabic, hydrothermal ZnO	~50 μm film thickness	*S. aureus*, *E. coli*, *B. subtilis*	35 °C, 54% RH	>17 days	Maintained firmness, sugars, TA	[145]
Green bell pepper	CS, CS NP, thyme essential oil (TEO) NP	CS NP: 6–20 nm and 80–900 nm; TEO-CSNP: 80–400 nm and 500–2000 nm. Coating thickness: 68.24 μm	*Pectobacterium carotovorum*	10 °C, 89% RH	12 days	Lowest CFU/disease incidence at 15% CS NPs; maintained weight loss, firmness; ↓CO_2_ production	[146]
Mango	Pectin (pequi mesocarp), ZnO NP	-	*Staphylococcus aureus*, *Pseudomonas aeruginosa*, and *Salmonella* Enteritidis	(20 °C)	12 days	Slowed ripening	[147]
Button mushroom	Nano-CS, aloe vera, tomato seed protein hydrolysate (TPH: 0.5–1.5%)	-	-	4 °C	16 days	↓ physicochemical changes, browning, microbial spoilage; ↑ antioxidant activity, sensory acceptance (1.5% TPH optimal)	[148]
Enoki mushroom	Nano aloe vera, nano sodium alginate	Average particle size: nano aloe vera—452.9 nm; nano sodium alginate—489.8 nm	-	10 °C	21 days	↑ Phenolics, flavonoids, antioxidants; maintained bioactive quality	[149]
Beef	CS, cress seed gum		Total viable count, psychrotrophs, *Pseudomonas aeruginosa*, *Staphylococcus aureus*	(−2, 4, 8 °C)	18 days	↓ Lipid oxidation and microbial growth; ↑ antioxidant property	[150]
Mango	Corn starch, ZnO (0.5, 1, 1.5, 2 M)	Mean particle size at 1.5 M coating—21.59 nm	-	Room temperature	7 days	↓ Fruit senescence, fungal growth, weight loss; Preventing decay and maintaining sensory quality	[151]
Button mushroom	Guar gum, kefirn, *Murraya koenigii* berry extract	-	-	4 °C, 85–90% RH	8 days	↓ Weight loss, cap structure, TSS; delayed cap opening	[152]

TA—Titratable acidity; TSS—Total soluble solids; ZnO—Zinc oxide; CS—Chitosan; NP—Nanoparticles; ↑ indicates increase or enhancement; ↓ indicates decrease or reduction.

## Data Availability

No new data were created or analyzed in this study. Data sharing is not applicable to this article.
